# Self-Criticism in In-Work Poverty: The Mediating Role of Social Support in the Era of Flexibility

**DOI:** 10.3390/ijerph19010609

**Published:** 2022-01-05

**Authors:** José Antonio Llosa, Esteban Agulló-Tomás, Sara Menéndez-Espina, María Luz Rivero-Díaz, Enrique Iglesias-Martínez

**Affiliations:** 1 Department of Social Education, Padre Ossó Faculty, University of Oviedo, 33008 Oviedo, Spain; iglesiasenrique@uniovi.es; 2 Department of Psychology, University of Oviedo, 33003 Oviedo, Spain; estomas@uniovi.es (E.A.-T.); mariluzriverodiaz@gmail.com (M.L.R.-D.); 3 Faculty of Humanities and Social Sciences, University Isabel I, 09003 Burgos, Spain; sara.menendez.espina@ui1.es

**Keywords:** in-work poverty, precarious work, social support, social exclusion, coping strategies, self-criticism

## Abstract

In-work poverty reflects situations of income below the poverty threshold among employed people, involving a deterioration of wellbeing. The International Labour Organization prioritises this situation, which in countries such as Spain, Germany or Italy reaches rates of 11.8%, 10.6% and 11.8%, respectively. Within a context of flexibility, the occupational situation tends to be understood as an individual responsibility, which is why this study analyses the increase in self-criticism in these situations, and the role of social support in this relationship. The mediation of social support in the manifestation of self-criticism among people experiencing in-work poverty is analysed. The participants were 1430 employed people, grouped into those in a situation of poverty and those who are not. The results show that people in a situation of in-work poverty present a higher score in self-criticism and lower in social support. Social support is a mediating variable that prevents the manifestation of self-criticism. Lastly, a gender analysis shows that women experience this relationship more intensely. These findings enable a critical assessment of the activation policies that only take an individual approach. As an alternative, we propose strengthening interventions that foster social support, particularly among women.

## 1. Introduction

International bodies, such as the International Labour Organization, stress the priority of addressing situations of in-work poverty in different European countries [1]. A progressive increase has been observed of a phenomenon which in Spain, according to the European Union Statistics on Income and Living Conditions (EU-SILC) database, remains at 11.8% even after the most critical years of the economic crisis that started in 2008. In 2020 Spain was the fourth country of the European Union 28 (EU28) after Romania (14.9%), Turkey (12.9%) and Luxembourg (11.9%) in terms of in-work poverty. This situation has intensified over the last decade, and should not be considered something that affects specific countries, but a structural element of the current labour market [2]. Therefore, it is of widespread relevance in different European economies: for example, in Germany it was at 10.6% in 2020; in France—7.5%; Italy—11.8% in 2019, or in Greece at almost 10% (9.9%).

In order to understand this trend, it is necessary to look at the characteristics of the labour market. The European Commission, in its employment activation policy, points out the importance of labour flexibility [3,4]. In theoretical terms, labour market flexibility means that people could regularly change jobs, but the ease of hiring coupled with the demand of companies to attract talent would lead to a mobile and dynamic labour market. At the same time, employees would have sufficient labour opportunities. However, this rhetoric ignores the fact that the labour market in western democracies is narrowing due to the progressive automation and relocation of production, and the increase in job opportunities in precarious sectors such as the services sector. This results in a loss of labour demand, which has a large impact on low-skilled jobs [5,6,7].

Thus, the European labour market is seeing a progressive shift towards job insecurity as a result of the combination of the deregulation brought by the paradigm of flexibility. In countries such as Spain, this deregulation manifests itself in a succession of labour reforms that find their ultimate expression in the Spanish labour reform brought by Royal Decree-Law 3/2012, of 10 February, which creates easier conditions for dismissal. At the same time, involuntary part-time employment rates have progressively risen, reaching 23.6% in 2019 for the EU-28. Countries with Mediterranean welfare models, where job stability was more protected in legal terms and legitimised in cultural terms, are seeing a rapid transformation [8,9,10]. Spain shifted from 33.3% to 52.2% of involuntary part-time employment between 2007 and 2020. It affects women substantially more, reaching 21.6% of part-time employment in 2019, in comparison to 8.8% among men. At the same time, the temporary employment rate of the EU-28 rose to 15% at the most intense moments of the economic crisis (2017), but remains a structural phenomenon above 14% in the most recent records. Again, the countries with a Mediterranean model are the most affected: Spain (21.7%), Italy (16.2%) or Greece (15.3%). These data not only explain the evolution of in-work poverty, but also its chronic nature [11,12,13].

### 1.1. Complexity and limitations of the Concept

The first complexity of the phenomenon of in-work poverty is of a conceptual nature. The data shown above are extracted from official European statistics sources (EU-SILC). In this case, in-work poverty is defined as a condition: “In-work at-risk-of-poverty rate refers to the percentage of persons in the total population who declared to be at work (employed or self-employed) who are at-risk-of-poverty (i.e., with an equivalised disposable income below the risk-of-poverty threshold, which is set at 60% of the national median equivalised disposable income (after social transfers)” [14] (p. 1). In the methodology of the EU-SILC it is specified that those who have been employed for at least 7 months during the previous year are excluded, and the age range contained in the main dataset is from 18 to 59. However, this definition has some significant limitations. The first of them is that it is considered a phenomenon of a familial nature [15].

The poverty rate sets the threshold at 60% of the median household income according to the AROPE indicator [16]. The gender pay gap indicates that there is a difference in remuneration between men and women, materialising in all OECD countries except Hungary [17]. Faced with these data, authors such as Crettaz and Bonoli [18] point out that the breadwinner model is still in effect, according to which the main source of income in a household headed by a heterosexual couple tends to be the male’s salary. In fact, it is observed that in-work poverty is better explained by the fact that in a household there are two sources of income, than by association with the low salaries of the breadwinners [19]. Thus, there is the risk of hiding the low salaries received by women in the statistics collected, and this can be observed in the high figures of in-work poverty among single-parent households [17,20,21].

Single-parent households record an at-risk-of-poverty rate in countries such as Spain that is close to 50%, while more than 80% of these families are headed by women [16]. Research shows high rates of employability among these women, with low salaries and precarious working conditions [21]. With this situation, Marx et al. [19] declare that an increase in the minimum salary, by itself, is not an effective measure against in-work poverty, as what is necessary is a reduction of the gender pay gap that guarantees standard working conditions for women and men.

Likewise, young people experience a widespread situation of precarious employment, becoming a factor of vulnerability regarding in-work poverty [3]. The complexity of accessing the labour market for young people, as well as the low quality of the positions achieved, conditions youth emancipation or the ageing of the European population. The countries where the employment situation of young people is more lacking is where these phenomena have the biggest impact [2].

On the other hand, the need to be employed for a period of seven months during the previous twelve in order to achieve the consideration of in-work poverty hides the situation of households with low employment intensity. These households are not only characterised by highly intermittent employment conditions, but also by the fact that the jobs they have access to tend to be precarious [3].

Following the recommendation of authors such as Halleroed et al. [2] or Marx and Nolan [22], this research paper adapts the conceptualisation of in-work poverty. The measurement of relative poverty proposed by the AROPE indicator is maintained, establishing the threshold at 60% of the national median income after social transfers. However, the seven-month employment during the last year criterion is not contemplated, considering in-work poverty affecting any person who while in employment does not receive sufficient income to bring them above the poverty threshold.

### 1.2. In-Work Poverty and Health

The condition of in-work poverty has an impact on the wellbeing of people. Thus, a deterioration of mental and physical health related with this form of precariousness has been observed [23]. A relationship has also been shown between the deterioration of perceived overall health and poverty [24,25]. This relationship is stronger in the case of women [24]. Similarly, factors related to occupational health are affected in a context of in-work poverty. Bearing in mind that in-work poverty is linked to more precarious positions, characteristic of micro-companies, the poor development of occupational health and safety in these contexts leads to a deterioration of wellbeing [23,26]. What is more, despite the fact that the condition of poverty negatively affects health, these people tend to use healthcare systems less. In countries where healthcare is not universally covered by public systems, this deficit in the use of primary care is mainly due to economic factors [26]. For example, in Canada a lesser degree of use of dental care—not fully covered in the national healthcare system—is observed. [27]. However, in countries where the healthcare system offers universal coverage there is also less use of them by people experiencing in-work poverty [23]. This is explained by the fact that these people cannot afford to abandon their employment obligations, or risk losing their jobs. This situation leads to a vicious circle between the condition of in-work poverty and deteriorating health.

The scientific literature on social exclusion shows that a situation of poverty is linked to mental health symptoms, as well as increased consumption of psychopharmaceuticals [28]. Similar results can be observed with a situation of precarious work in a broader sense [29]. When trying to limit the phenomenon studied explicitly to in-work poverty, there is not a large number of studies that specifically address this matter. Attempting to mention some of them, Moon and Sangjun [30] detect a higher prevalence of depression in situations of in-work poverty. Similarly, they link this employment situation with an increase in the consumption of alcohol, which intensifies the mental disorder experienced. As for the conditions of this relationship, they point out the inability to afford housing-related expenses, and show that it clearly affects women more than men.

A study carried out in Spain presents the same conclusion as Moon and Sangjun [30], pointing out that the general state of mental health of people experiencing in-work poverty is the same as those who are unemployed, while in both cases there is a deterioration with respect to the people who are in a normalised employment situation [31]. Likewise, chronic stress is linked to situations of in-work poverty given the uncertain living conditions that these families experience [31,32].

### 1.3. Social Support and Wellbeing in Contexts of Precariousness

In conditions of social exclusion, social support is a variable that is highly affected [33,34]. Perceived poverty is explained in a trend towards situations of isolation of the population [29]. This factor is important, not only because social support is a fundamental variable to explain overall wellbeing and mental health, but because it is an element to be considered in the chronification of situations where there is a risk of social exclusion [35,36].

In Hong Kong experiments are being carried out with active health programs among people in a situation of in-work poverty, which include accompaniment and social support as one of the elements. A positive impact on this population has been observed [37]. Likewise, Moon and Sangjun [30] point out the importance of social support to protect the health of the people in this situation of precariousness. Traditionally, the professional environment had been described as a space for strengthening social support. However, in contexts of precariousness and uncertainty, this function disappears [31,33,34].

The situation of social exclusion risk and loss of social support also has an impact on the political and ideological dimension. Studies have shown that people in a situation of in-work poverty, due to their condition of social exclusion, tend to experience disengagement with respect to the institutions, which puts at risk social cohesion and coexistence between territories [7,15,38,39].

### 1.4. Coping Strategies and Labour Activation

What has been explained up until now contrasts with the European Union’s employment policy strategy, highly focused on individual employment activation and which prioritises economic production capacity over the population’s wellbeing [40]. In fact, an incongruence can be detected in this approach, as in-work poverty figures rose in the European Union even during times of economic growth before the economic crisis [41]. Thus, and regardless of whether the welfare state regime is more or less redistributive, in general terms the labour policy in terms of in-work poverty has been characterised by two features: subsidies allocated for poverty risk and social exclusion are conditional upon the employment activation of the beneficiaries, and social protection systems are fundamentally employment activation devices [41]. On the one hand, this means that the policies developed are not being very effective, and on the other, they make the people in this situation individually responsible [42,43,44]. This approach involves risk, as authors such as Skilling and Tregidga [45] showed that discourses that focus on economic growth as a strategy to eradicate poverty legitimise situations of inequality.

Vander Elst et al. [46] explain how precarious work conditions produce chronic stress, which leads to learned helplessness. Therefore, the coping strategies developed by people in a situation of precarious employment tend to be evasive, individualistic and particularly focused on self-criticism (cognitive strategies focused on criticising oneself and blaming oneself) in order to cope with the process they are experiencing [29,47]. As pointed out by these authors, self-criticism never leads to the solution of the problem experienced by the person, but to an emotional assessment of the same that worsens the situation they experience.

### 1.5. This Study

In previous sections it is observed that the conditions of in-work poverty lead to a deterioration of wellbeing. Social support is a protection strategy for the people in this situation, but is limited in conditions of precarious work [48]. Instead, people in a situation of precariousness tend towards self-criticism in personal terms to cope with the situation they experience. In view of these findings, the research question that this study proposes is how the variables of self-criticism and social support relate to each other among people in a situation of in-work poverty. To do this, three hypotheses are designed to be tested.

The hypothesis is that the condition of in-work poverty leads to a greater probability of using self-blame coping strategies, such as the self-criticism strategy (Hypothesis 1). Knowing the important role that social support has for people experiencing in-work poverty, the hypothesis is proposed that social support is a mediating variable of this relationship (Hypothesis 2). Thirdly, the hypothesis is that social support will have a more important role as a mediating variable among women than among men (Hypothesis 3).

## 2. Materials and Methods

### 2.1. Participants

Quota and convenience sampling were conducted. The target sample of this study was comprised of men and women in employment at the time of answering the questionnaire. All of the participants work and reside in Spain. The sample of volunteers with online participation was collected during the year 2019. After removing incomplete answers and those that did not meet the previously mentioned criteria (employees at the time of responding to the survey; between 18 and 65 years old, and residents in Spain), the study used a total sample of 1429 people. Of these, 772 were women with an average age of 36.02 years (SD = 11.58) and 658 men with an average age of 34.28 years (SD = 12.61).

Of the participants, 1124 (78.6%) were employed with income above the poverty threshold and 305 (21.4%) people were in a situation of in-work poverty. Of the people in a situation of in-work poverty, 193 were women and 112 men. The characteristics of the sample are detailed in Table 1.

The proportion between employees in poverty and employees without risk of poverty in the sample is close to the proportion in the Spanish labour market. In 2019, the Spanish employment poverty rate was 12.7%, compared to 87.3% above the poverty line. As it is a convenience sampling. The characteristics and size of the sample focused on staying close to this proportion between groups.

### 2.2. Procedure

The collection of data was carried out in digital format and individually. Informed consent was obtained from all subjects involved in the study. There are also informed of its voluntary nature, that the data would only be used for research purposes, and that all information collected was anonymous. They did not start completing it without having first given explicit, written consent. The collection of data was carried out with the Survey Monkey tool, which offers the necessary guarantees of data encoding to ensure the anonymity of the participants. This study was conducted in accordance with the Declaration of Helsinki, and the protocol of Ethical Committee of the Psychology Department of the University of Oviedo.

### 2.3. Instruments

A battery of questionnaires was administered to measure perceived social support, the self-criticism coping strategy and the social and professional conditions of the participants.

Perceived social support: measured with the MOS scale [49], validated for the Spanish population with a reliability close to 1 [50]. This scale is comprised of 19 5-point Likert-type items (measurement of perceived social support), and an open question that measures the number of family and friends whom the people surveyed considered to be support figures (social support network). This study analyses the total score of perceived social support offered by Likert-type items. The higher the score, the greater the perception of support according to the scale. We also used the measurement of the size of the social support network offered by the open score of the questionnaire.

Self-criticism: This is one of the coping strategies measured by the CSI (Coping Strategies Index) [51], validated for its application in the Spanish context [52]. The subscale of measured self-criticism (α = 0.94) is comprised of 9 5-point Likert-type items. For this subscale, the higher the score, the greater the tendency towards using the self-criticism coping strategy.

Social and professional conditions: the last part of the battery administered is formed by a set of items that seek to detect information related to the occupational situation of the interviewees; their at-risk-of-poverty and/or social exclusion risk by means of the items of the AROPE (At Risk of Poverty or Exclusion) indicator; as well as general sociodemographic information to define the characteristics of the sample studied.

### 2.4. Analysis

In order to examine the relationship between the variables analysed, the entire sample was subjected to a correlational analysis of the variables (CI 95%): condition of in-work poverty, self-criticism strategy (CSI self-criticism factor), score in perceived social support (MOS) and size of support network, also with (MOS). A descriptive analysis was then carried out which compared with the *t*-test (CI 95%) for independent samples (in-work poverty situation and not) the perceived social support variables, size of the network and self-criticism strategy. This *t*-test analysis was carried out for the total sample, as well as for women and men separately.

In order to respond to the objectives of this study a mediation model was designed through the macro PROCESS version 3.5.3 by SPSS (IMB, New York, NY, USA) designed by Hayes [53]. To test the first hypothesis the total effect model of the dichotomous in-work poverty variable (IV) was calculated with the macro, where value 0 is the condition of employment without risk of poverty and 1 is in-work poverty, on the self-criticism strategy variable (DV) (CI 95%).

Then the second hypothesis was tested with a succession of simple mediation analyses performed with model 4 of PROCESS. In the first of the analyses the indirect effect of the in-work poverty variable (IV) on the self-criticism strategy variable (DV) was calculated through the mediation of the perceived social support variable (M). In the second, the mediating variable was the size of the support network. These analyses were carried out for the entire sample, through 10,000 samples of bootstrap. From the resulting interval it is assumed that the indirect effect is significant (CI 95%) when the value 0 is not included within its limits.

In order to test the third hypothesis, the same disaggregated analysis between men and women was carried out. A multiple mediation analysis with chained variables was performed. With model 6 of PROCESS the indirect effect of the independent variable (IV), in-work poverty, on the dependent variable (DV), self-criticism strategy, was calculated through the sequencing of the two variables with respect to the social support network analysed previously and dealt with in this order: size of the social support network as the first mediating variable (M1) and perceived social support as the second mediator (M2). Again, the analysis was performed with 10,000 samples of bootstrap and CI 95%.

## 3. Results

The descriptive analyses show a significant correlation for the total sample between the variables involved in the study (Table 2). The correlation is positive between the perceived social support score and the size of the support network (r = 0.28, *p* < 0.01), while it is negative with self-criticism (r = −0.17, *p* < 0.01). Self-criticism is also negatively correlated with the size of the support network (r = −0.11, *p* < 0.01). This indicates that good social support is related with a greater tendency towards self-criticism, as maintained by the theoretical proposal made. Likewise, a larger support network size is associated with higher scores of perceived social support.

As for the analysis of in-work poverty, the *t*-test for two samples shows significant differences between people in a situation of in-work poverty and with employment above the poverty threshold for the score in self-criticism (t = −4.17, *p* < 0.01), as well as for the two social support variables: perceived social support (t = 5.09, *p* < 0.01) and size of the social support network (t = 2.62, *p* < 0.01). Observing the averages, the theoretical proposal stands, as with a situation of in-work poverty there are worse scores in self-criticism (M = 12.63, SD = 4.9) than in the group whose employment generates income above the poverty threshold (M = 11.32, SD = 4.56). This same relationship can be observed with the averages of perceived social support and size of the support network. When replicating the analyses of the *t*-test with men and women separately, the relationships remain significant and in the same direction as that shown, except in the case of the size of network. In the case of men, no statistically significant differences are observed between the group experiencing in-work poverty and those above the poverty threshold with respect to the size of the support network (t = 1.18, *p* = 0.24). These results indicate a gender difference, which is congruent with the results that will be shown later with the mediation analysis related to the third hypotheses of the study (Table 3).

**Figure 1:**
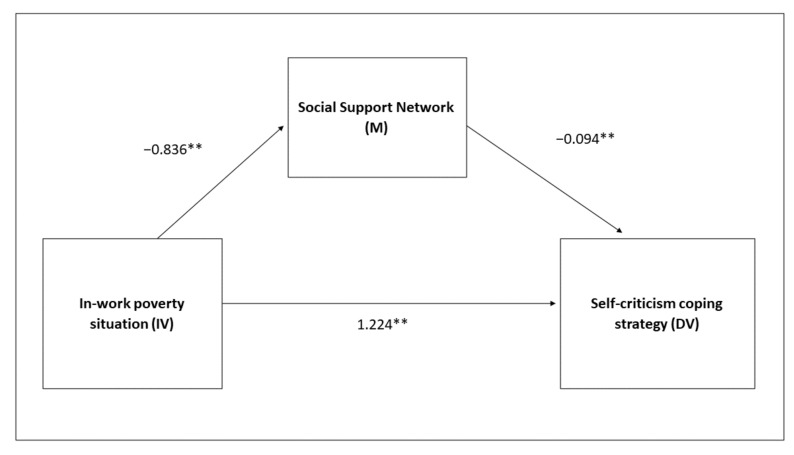
Simple mediation model with the mediating variable being size of the social support network. Joint sample of men and women. ** *p* < 0.01.

Then, we continued with the mediation analyses that provide a response to the three hypotheses put forward. The total effect of the in-work poverty condition variable (IV) on access to the self-criticism coping strategy (DV) was studied, testing Hypothesis 1. It is confirmed that the in-work poverty condition in a sample composed of men and women is associated with greater access to self-criticism (B = 1.3, *p* = 0.001) (Figure 1).

**Figure 2:**
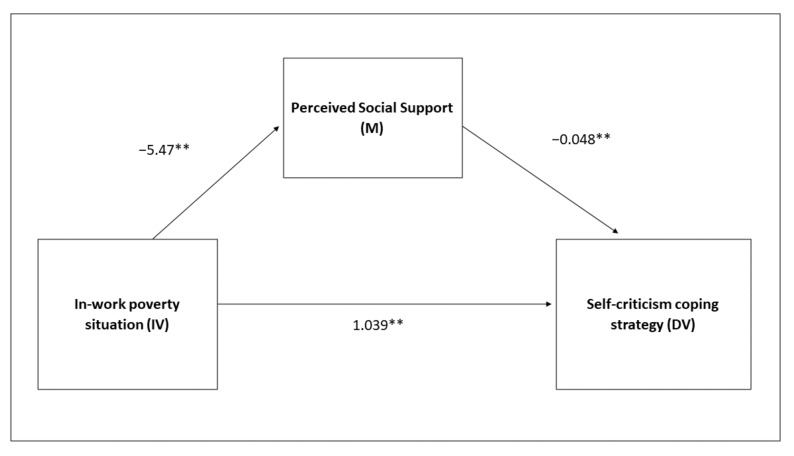
Simple mediation model with the mediating variable being perceived social support. Joint sample of men and women. ** *p* < 0.01.

There is also confirmation of the second hypothesis. The measurement of social support proposed as a mediator is analysed with the standardised MOS test [49,50], which provides two scores: a score on the number of people who make up the respondent’s support network and another with the social support this person perceives. Analysing the indirect effect of each one of these variables in the relationship that is established between the in-work poverty condition (IV) and self-criticism (DV), it is shown to be relevant in both cases. People in a situation of in-work poverty have a greater probability of accessing negative strategies, in this case self-criticism, and this relationship is mediated by the size of the social support network (M) (B = 0.08, SE = 0.04; 95% CI = 0,02;0,16), as well as by the perception of social support (M) (B = 0.26, SE = 0.07, 95% CI = 0.13;0.41). In addition, it was observed that the situation of in-work poverty is related to worse social support scores. Again, this relationship is clear in both the size of the network (B = −0.836, *p* = 0.01), and as regards the perception of social support (B = −5.475, *p* = 0.01) (Figure 2).

The direct effect of the situation of in-work poverty and the self-criticism coping strategy is also significant in both cases: when the mediation of the support network size is analysed in the model (B = 1.04, *p* = 0.01) and when the mediation of the perceived support network is dealt with (B = 1.302, *p* = 0.01). This indicates the presence of other variables, in addition to the social support ones, that are relevant in this case (Table 4).

As for hypothesis 3, men and women were studied separately in order to detect differences in the mediation that is tested. As for the relationship between the condition of in-work poverty and access to self-criticism strategies, what has been shown previously is still maintained. Both in the case of women (B = 0.81, *p* = 0.034) and men (B = 2.123, *p* = 0.001), the condition of in-work poverty affects access to self-criticism coping strategies. Having confirmed this, we analysed whether social support was mediating the relationship in this case.

**Figure 3:**
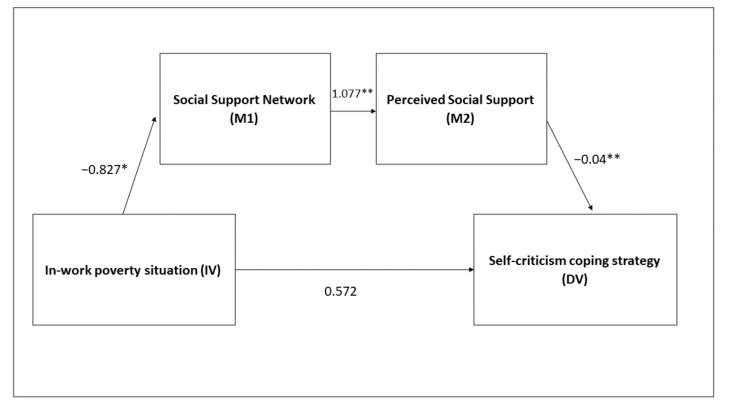
Mediation model with women. * *p* < 0.05, ** *p* < 0.01.

First, the sample of women was studied, including both moderating variables studied in sequence: first the support network and then the perceived social support. This condition is designed in this way because having a wide support network is not a guarantee of perceived support, though it does have an important relationship (r = 0.281, *p* = 0.001). It is concluded that women in a situation of in-work poverty with a smaller support network will have a tendency to manifest lower perceived social support, which conditions a higher probability of developing self-criticism strategies (B = 0.045; SE = 0.02; CI = 0.004; 0.084). When observing the direct effect of the situation of in-work poverty and the self-criticism strategy, it is not significant (B = 0.573, *p* = 0.13), which indicates that social support is a necessary and effective measure to control unproductive strategies in the case of women in a situation of in-work poverty (Figure 3).

**Figure 4:**
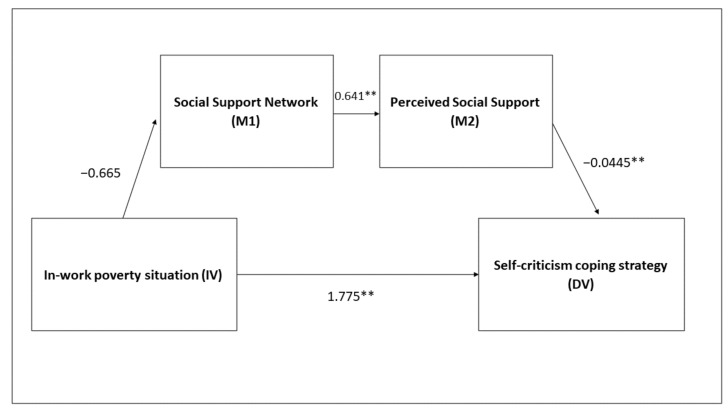
Mediation model with men. ** *p* < 0.01.

On the other hand, this does not happen in the case of men. Analysing the sample of men, it was observed that the mediation model with the sequence of mediating variables, support network and perception of support, is not significant (B = 0.02, SE = 0.02, CI = −0.013; 0.064). This is determined because, in their case, perceived social support is a relevant mediating variable in the relationship between in-work poverty and self-criticism (B = 0.29, SE = 0.12, CI = 0.096; 0.561), but not the size of the support network (B = 0.04, SE = 0.04, CI = 0.12; 0.65) (Figure 4) (Table 5).

## 4. Discussion

### 4.1. Self-Criticism Strategy among People in a Situation of In-Work Poverty

**Hypothesis** **1**. *The condition of in-work poverty leads to a higher probability of using self-blame coping strategies, such as the self-criticism strategy.*

The first hypothesis of the study is accepted according to the results shown. The condition of in-work poverty involve a higher probability of accessing self-blame coping strategies, such as self-criticism. This concurs with Vander Elst et al. [46], by supposing that the condition of precarious employment involves more negative coping. Menéndez-Espina et al. [48] pointed out that the self-criticism coping strategy is particularly relevant to wellbeing in the case of women. However, in this study we show that the condition of in-work poverty has a similar behaviour both in the case of women and in that of men.

Previous studies have observed the manifestation of coping strategies in different contexts of precariousness, such as migrants in Denmark [54] or healthcare workers in Brazil [55]. In the case of migrants, they use strategies to rationalise the situation of precariousness they are going through. In the case of healthcare workers exposed to situations of precariousness, there have been strategies that are an attempt to improve their working conditions. Both cases share two points in common: first, an attempt to put into practice strategies to change the situation they are going through; second, that in none of them the strategies achieved the hoped-for success. Leading to the chronification of this situation of precariousness over time or increasing its intensity, coping strategies become more unproductive when exposed to stressors of growing relevance [46]. In this sense, the results shown point to the situation of in-work poverty, a very intense situation of precariousness. It is observed that people in a situation of in-work poverty continue attempting to put coping strategies into practice, but they are clearly unproductive: focused on an emotional assessment such as self-criticism, and therefore with poor abilities of transforming the environment.

Self-criticism is related with hyperreflexivity, one of the most common symptoms of depression. In this sense, the results shown increase the deterioration of mental health among people in a situation of precariousness, something which in the case of in-work poverty can be verified in previous studies [31,48].

In contrast with the internal assessment that people in a situation of in-work poverty carry out, the literature shows that in-work poverty responds to structural and collective factors [3]. Studies mention as structural factors income, the quality of job market offerings, flexibility, the gender gap or being young [2,20]. However, there is not a proven relationship between the condition of in-work poverty and personal traits. Why, then, is the assessment of the people who experience it internal? Working relationships understood in individualised terms tend psychologically to generate individual responsibility over lived experience [42,56]. The intervention models based on employment activation—occupational training and active job seeking—are poorly effective in terms of results [41], but generate a psychologising narrative with an impact on employment relationships [44]. Thus, precarious work is not only negative for wellbeing in terms of the impossibility of covering material needs [57], but also the experience itself of the situation as self-criticism.

Korean-born German philosopher Han [58], in his work *Psychopolitics: Neoliberalism and New Technologies of Power*, developed this idea of individual psychological examination within the social context. This examination makes people permeable in their experience of precariousness, and it also supports the psychological discipline of a more individual perspective [59]. With these results, one of the first conclusions of the study explains that the tendency towards self-criticism is not a personal trait of the subjects that predicts less success. The conditions of exclusion—such as in-work poverty—generate a framework of defencelessness that is prone to evaluating these experiences in terms of self-criticism.

### 4.2. Social Support as a Mediating Variable of Self-Criticism

**Hypothesis** **2**. *Knowing the important role that social support has for people experiencing in-work poverty, social support is a mediating variable of this relationship.*

The second hypothesis of the study is also accepted. This hypothesis states that knowing the important role that social support has for people experiencing in-work poverty, social support is a mediating variable of this relationship between in-work poverty and self-criticism.

Firstly, it is observed that social support is greater among people in employment than among those experiencing in-work poverty. Authors such as Park et al. [60] show that employment is a source of social support; however, this relationship is only maintained in quality employment. For both men and women, this relationship is observed in the two measurements of social support taken in the results with the MOS scale: both perceived social support and the size of the support network. Social support is related with measurements of wellbeing, such as mental health, meaning that it is a protection factor [61]. On the other hand, social support mediates the relationship between precariousness and the self-criticism strategy. Thus, people who have greater social support have a lower tendency towards self-criticism. In this sense, community intervention measures—which foster social support—are more effective in contexts of in-work poverty than individualistic or conditional measures [37,41].

Social support is also a useful tool to predict professional success, in that it facilitates employment opportunities [62]. However, we have observed that the situation of in-work poverty, which implies social exclusion, reduces the support network [29,31]. This creates a vicious circle which chronifies the condition of precariousness and has a negative impact on the wellbeing of the people affected by these situations.

### 4.3. Gender Differences in In-Work Poverty

**Hypothesis** **3**. *Social support will have a more important role as a mediating variable among women than among men.*

Lastly, the literature on in-work poverty indicates that one of the factors that explain this phenomenon is related to gender [17,20,21]. Thus, women have a greater likelihood of going through a situation of in-work poverty throughout their professional career than men. This study also confirms its third hypothesis, which maintains that social support will have a more relevant role as a mediating variable among women than among men (Hypothesis 3).

Firstly, although the measurement of perceived social support shows differences between people in employment above and below the poverty threshold, this does not happen in the case of the size of the support network. Men do not show differences in this measurement. This means that, regardless of the situation of in-work poverty, men have a higher likelihood of maintaining a broad support network. In this sense, the breadwinner model logic still stands among men [18], as they continue to maintain a support network that is independent of their condition of precariousness. However, women in a situation of social exclusion show a greater tendency towards isolation, as shown in the research by Dahlberg et al. [63]. It should be remembered that one of the characteristics of the patriarchal social model is that men lead a more public life, while women lead a more private life related to caring tasks [64].

This idea also has implications in the multiple mediation model designed, with both measurements of social support done separately. Among women, the mediation role of social support is more relevant to explain self-criticism, which leads to the conclusion, in line with the literature, that the situation of in-work poverty is not only more common among women [24,65], but has a greater impact on wellbeing as there is not such a broad support network available. Similar conclusions have been obtained in recent studies on gender and mental health. Kendler et al. [66] showed that there is a stronger link between the absence of social support and depressive symptoms in women than in men.

### 4.4. Limitations and Future Research

The basic premise is that this paper analyses a very specific condition of precariousness, which is the situation of in-work poverty. Therefore, it would be necessary to analyse whether the mediating relationship of social support between the situation of in-work poverty and self-criticism is maintained with other forms of precarious work. On the other hand, in-work poverty implies a situation of social exclusion, but it is not the only form of social exclusion of which we are aware. In fact, most people find themselves in a situation of social exclusion because they are in a situation of unemployment and long-term unemployment. It would be necessary to also analyse whether the conclusions of this study are applicable to other forms of social exclusion different to that analysed.

In the methodological section, it would be necessary to replicate studies like this one with probability sampling. This allows a greater capacity for generalisability of conclusions than convenience studies. For this reason, it would be important that data panels, such as EU-SILC, include brief psychometric scales of mental health or social support that allow these approximations.

## 5. Conclusions

As for the implications of this study, there are three issues to be addressed. The first is that it is not pertinent to present an analysis of coping strategies in individual cognitivist terms. The analyses of self-criticism presented show that it is a mainly person–world interactive phenomenon, and not only personal.

Secondly, we have observed that social support is a decisive variable for the expression of self-criticism. Therefore, the usual employment activation measures are not appropriate in these cases, and community interventions with a proven effect on the improvement of social support should be used [30,61,67].

Lastly, the gender analysis enables us to observe that the relational sphere is more deteriorated among women in a situation of in-work poverty. Therefore, the measures and policies to address in-work poverty must prioritise the social and professional gaps affecting women.

## Figures and Tables

**Table 1:** Sample participating in the study.

	In-Work Poverty		Age (SD)	N
	Not In-Work Poverty	In-Work Poverty		
Women	579	193	36.02 (11.58)	772
Men	545	112	34.28 (12.61)	658
Total	1124	305	35.22 (12.1)	1430

**Table 2:** Correlation between analysed variables.

	**1. Self-Criticism**	2	N
**2. Perceived Social Support**	−0.17 **		1430
**3. Social Support Network**	−0.11 **	0.28 **	

** *p* < 0.01.

**Table 3:** *t*-test for independent samples of in-work poverty and employment with income above the poverty threshold.

	Self-Criticism		Perceived Social Support		Social Support Network	
	M (SD)	T	M (SD)	T	M (SD)	T
**Total sample**						
In-work poverty	12.62 (4.91)	−4.17 **	74.32 (17.16)	5.09 **	8.15 (4.59)	2.62 *
Not in-work poverty	11.32 (4.56)		79.71 (14.64)		8.98 (4.58)	
**Women**						
In-work poverty	12.18 (4.86)	−2.12 *	75.08 (17.14)	3.34 **	7.88 (3.96)	2.22 *
Not in-work poverty	11.37 (4.52)		79.68 (14.87)		8.7 (4.64)	
**Men**						
In-work poverty	13.4 (4.92)	−4.4 *	72.77 (17.16)	4.5 **	8.62 (5.5)	1.18
Not in-work poverty	11.28 (4.6)		79.73 (14.4)		9.28 (5.4)	

* *p* < 0.05, ** *p* < 0.01.

**Table 4:** Results of the total effect of in-work poverty IV on self-criticism DV, and simple mediations with the size of the social support network and perceived social support as mediating variables.

	Effect	Boot SE	*p*	N (Men and Women)
**Total Effect:** (IV In-work poverty situation -> DV Self-criticism)	1.3 **	0.3	0.000	1430
	**Effect**	**Boot SE**	**CI 95%**	**N (Men and Women)**
**Indirect Effect:** (In-work poverty situation -> Social Support Network -> Self-criticism)	0.08 *	0.04	[0.02; 0.16]	1430
**Mediation:** (In-work poverty situation -> Social Support Network -> Self-criticism)	0.26 *	0.07	[0.13; 0.041]	

* CI = 95% provided the CI does not include the value 0 between the lower and upper limit. ** *p* < 0.01.

**Table 5:** Mediation model of multiple variables in men and women separately.

Indirect Effect		Effect	Boot SE	CI (95%)	N
**Women**					
	In-work poverty situation -> Social Support Network -> Perceived Social Support -> Self-criticism	0.4 *	0.02	[0.004; 0.084]	772
**Hombres**					
	In-work poverty situation -> Social Support Network -> Perceived Social Support -> Self-criticism	0.02	0.02	[−0.013; 0.064]	658
	In-work poverty situation -> Perceived Social Support -> Self-criticism	0.04 *	0.04	[0.12; 0.65]	658
	In-work poverty situation -> Social Support Network -> Self-criticism	0.29	0.12	[0.096; 0.561]	658

* CI = 95% provided the CI does not include the value 0 between the lower and upper limit.
